# Potentially inappropriate medication use among older adults with cognitive impairment and dementia attending primary care‐based memory clinics

**DOI:** 10.1002/alz.086936

**Published:** 2025-01-09

**Authors:** Rishabh Sharma, Linda Lee, Feng Chang, Tejal Patel

**Affiliations:** ^1^ University of Waterloo, Waterloo, ON Canada; ^2^ McMaster University, Kitchener, ON Canada

## Abstract

**Background:**

The use of potentially inappropriate medications (PIMs) in older adults with dementia and/or Mild Cognitive Impairment (MCI) has been associated with increased adverse events, drug‐related problems (DRPs), prolonged hospitalization, risk of falls, and increased length of stay. This study aimed to identify which explicit tool, Beers criteria 2023 or Screening Tool of Older Persons Potentially Inappropriate Prescriptions (STOPP) 2023, identifies more PIM use among older adults with MCI or dementia.

**Methods:**

A cross‐sectional study was conducted at a Multispecialty Interprofessional Team‐based (MINT) memory clinic. Patients with MCI or dementia were recruited between Jan and August 2023. Patient medical records were reviewed for PIMs using Beers Criteria 2023 and STOPP criteria 2023. Bivariate logistic regression analysis was employed to identify potential factors associated with the use of PIMs.

**Results:**

Overall, 44 participants were enrolled in the study, with a mean age of 80.2± 6.2 years. Among 44 patients, 36.4% (n = 16) patients had MCI, followed by one‐fifth of patients (n = 9) who had mixed dementia and 11.4% (n = 5) with vascular cognitive impairment. At least one PIM was identified in 47.7% (n = 21) and 27.2% (n = 12) of the study participants based on Beers' and STOPP’s criteria, respectively. Using the Beers criteria, 50 PIMs were found, with an average of 0.9 PIMs for each patient, while a total of 31 PIMs were identified using the STOPP criteria, with an average of 0.6 PIMs per patient. There was a significant association between ≥ 9 number of comorbidities and PIMs as per Beers criteria (OR = 8.4, 95% Confidence interval: 1.27‐ 55.39, P = 0.027). However, no statistically significant association was observed with PIMs as per STOPP criteria.

**Conclusion:**

The frequency of PIMs identified using Beers and STOPP criteria highlights the importance of identifying and addressing PIMs in this population. This study adds valuable insights to the progressing comprehension of medication‐related complexities in older adults living with MCI or dementia.

